# Discovery of a Novel Triazolopyridine Derivative as a Tankyrase Inhibitor

**DOI:** 10.3390/ijms22147330

**Published:** 2021-07-08

**Authors:** Hwani Ryu, Ky-Youb Nam, Hyo Jeong Kim, Jie-Young Song, Sang-Gu Hwang, Jae Sung Kim, Joon Kim, Jiyeon Ahn

**Affiliations:** 1 Division of Radiation Biomedical Research, Korea Institute of Radiological & Medical Sciences, Seoul 01812, Korea; hwanya85@kirams.re.kr (H.R.); hjkim@kirams.re.kr (H.J.K.); immu@kirams.re.kr (J.-Y.S.); sgh63@kirams.re.kr (S.-G.H.); jaesung@kirams.re.kr (J.S.K.); 2 Department of Research Center, Pharos I&BT Co., Ltd., Anyang 14059, Korea; kyn@pharosibio.com; 3 Laboratory of Biochemistry, Division of Life Sciences, Korea University, Seoul 02841, Korea

**Keywords:** tankyrase, tankyrase inhibitor, colorectal cancer, WNT/β-catenin pathway, combination therapy

## Abstract

More than 80% of colorectal cancer patients have adenomatous polyposis coli (APC) mutations, which induce abnormal WNT/β-catenin activation. Tankyrase (TNKS) mediates the release of active β-catenin, which occurs regardless of the ligand that translocates into the nucleus by AXIN degradation via the ubiquitin-proteasome pathway. Therefore, TNKS inhibition has emerged as an attractive strategy for cancer therapy. In this study, we identified pyridine derivatives by evaluating in vitro TNKS enzyme activity and investigated N-([1,2,4]triazolo[4,3-a]pyridin-3-yl)-1-(2-cyanophenyl)piperidine-4-carboxamide (TI-12403) as a novel TNKS inhibitor. TI-12403 stabilized AXIN2, reduced active β-catenin, and downregulated β-catenin target genes in COLO320DM and DLD-1 cells. The antitumor activities of TI-12403 were confirmed by the viability of the colorectal cancer cells and its lack of visible toxicity in DLD-1 xenograft mouse model. In addition, combined 5-FU and TI-12403 treatment synergistically inhibited proliferation to a greater extent than that in a single drug treatment. Our observations suggest that TI-12403, a novel selective TNKS1 inhibitor, may be a suitable compound for anticancer drug development.

## 1. Introduction

WNT/β-catenin signaling plays crucial roles in embryo development and tissue homeostasis. A recent analysis by The Cancer Genome Atlas (TCGA) revealed that 93% of colorectal cancers (CRC) have genetic alterations of the WNT signaling pathway, which have been identified as biallelic inactivation mutations of *APC regulator of WNT signaling pathway* (*APC*), a negative regulator of β-catenin/CTNNB1, or activating mutations of *CTNNB1* in approximately 80% of the cases [1]. Canonical WNT signaling is activated when Wnt ligands bind to the Frizzled (Fzd) receptor. In the absence of Wnt ligands, β-catenin is scaffolded by the ‘destruction complex’ consisting of AXIN, APC, casein kinase 1 (CK1), and glycogen synthase kinase 3β (GSK3β). β-catenin, which is sequentially phosphorylated by CK1 and GSK3β, is ubiquitinated by E3 ubiquitin ligase (β-transducin repeat-containing protein; β-TrCP) and degraded by the 26S proteasome. In the presence of Wnt ligands, Fzd and LRP5/6 receptors are activated, and disheveled (DVL) polymers are formed. The complex binds to AXIN, GSK3, and CK1 and inhibits GSK3, leading to β-catenin accumulation [2]. Accumulated β-catenin translocates to the nucleus and binds to the T-cell factor/lymphoid enhancement factor (TCF/LEF) transcription factor, triggering upregulation of target genes, such as *MYC* and *AXIN2* [3]. However, loss-of-function of APC in the β-catenin destruction complex or gain-of function of CTNNB1 leads to aberrant accumulation of β-catenin and expression of its target genes. The inhibition of WNT/β-catenin signaling has known as an important therapeutic target over several decades. Despite of tremendous efforts in the development of inhibitors for WNT/β-catenin signaling, no drugs for clinical use have been promising yet.

The tankyrase protein has been proposed as a way to inhibit β-catenin signaling. Tankyrase (TNKS/TNKS1) and tankyrase 2 (TNKS2) (also known as poly (ADP-ribose) polymerase 5A (PARP5A) and 5B (PARP5B)) are members of the poly (ADP-ribose) polymerase (PARP) family of proteins and have PARP catalytic domains [4,5]. The TNKS1/2 proteins are important in mitosis regulation, telomere maintenance, and canonical Wnt pathway regulation [6,7,8]. The *TNKS1* and *TNKS2* genes have overlapping functions, based on the survival of *TNKS1* or *TNKS2* knockout mice and embryonic lethality in double knockout mice [9]. Therefore, the functional differences between TNKS1 and TNKS2 remain unknown. The structure of TNKS comprises five ankyrin (ANK) repeats, a sterile alpha motif (SAM), and a carboxy-terminal PARP catalytic domain [4,10]. TNKS1/2 sequentially add multiple ADP-ribose moieties to target proteins using NAD+ as a substrate. The ANK domain of TNKS binds to the target substrates and causes ADP-ribosylation of the substrates [11]. Several target proteins for TNKS have been identified, including telomere repeat binding factor 1 (TRF1), axis inhibitory protein (AXIN), phosphatase and tensin homolog (PTEN), nuclear mitotic apparatus protein (NuMA), insulin-responsive amino peptide (IRAP), 182-kD tankyrase-binding protein (TAB182), formin-binding protein 17 (FBP17), CBP80/CBP20-dependent translation initiation factor (CTIF), and peroxiredoxin II (PrxII) [7,10,11,12,13,14,15]. Among them, AXIN is a component of the β-catenin destruction complex and can act as a suppressor of the canonical Wnt signaling pathway by anchoring β-catenin and preventing its translocation to the nucleus. TNKS induces AXIN degradation and stabilizes β-catenin, upregulating the expression of Wnt/β-catenin target genes [16,17]. Thus, the development of TNKS inhibitors has been challenged by inhibition of Wnt/β-catenin signaling with stabilizing the negative regulator AXIN.

Recently, TNKS inhibitors such as XAV939, IWR-1, G007-LK, and NVP-TNKS656 have been reported to show inhibition of cell proliferation in β-catenin-dependent CRC cells with APC mutations [7,18,19,20,21]. Since E7449, a dual inhibitor of PARP 1/2 and TNKS, is the only drug currently under clinical trials, TNKS inhibitors need to be continuously developed and studied as anticancer drugs to elucidate the biological aspects of cancer cells [22]. Herein, we describe the identification of a novel small-molecule selective TNKS inhibitor, TI-12403, and suggest that TI-12403 is a potent TNKS candidate for the development of a novel TNKS inhibitor.

## 2. Results

### 2.1. Identification of TI-12403 as A Novel Potential TNKS Inhibitor

To develop small molecules that inhibit TNKS1, we designed and synthesized 17 chemical compounds based on the crystal structure of TNKS1-based virtual screening (Supplementary Schemes S1 and S2; Supplementary Figure S1). TNKS enzyme activity of the compounds was measured using a cell-free TNKS enzyme assay system. Eight compounds (TI-12402, -12403, -12405, -12407, -12409, -12410, -12412, and -12417) showed the highest TNKS inhibitory activity at 1 μM (Supplementary Table S1). 

**Figure 1:**
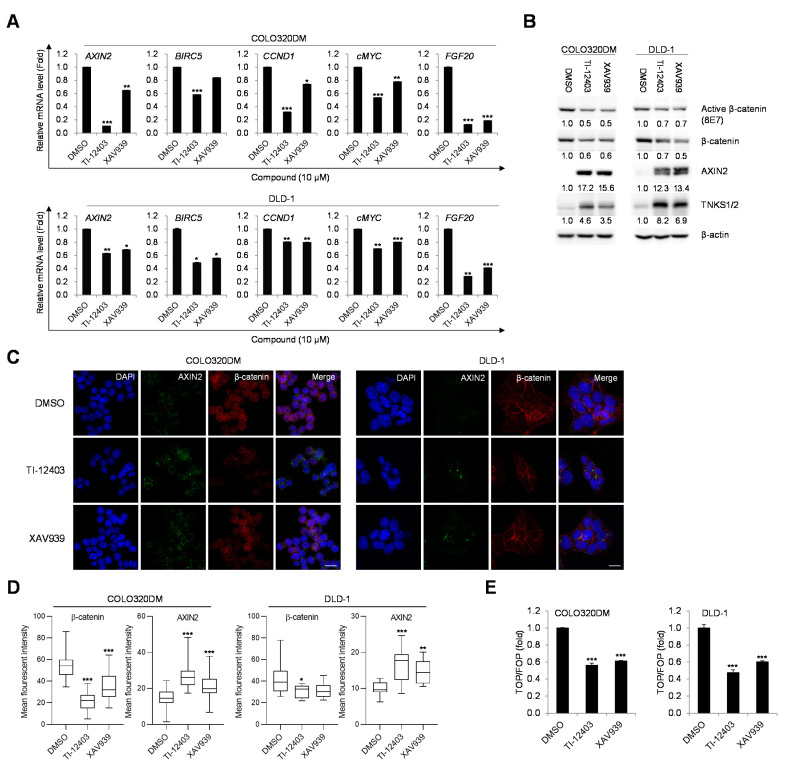
TI-12403 stabilizes AXIN2 and downregulates β-catenin signaling in COLO320DM and DLD-1 cells. Human colorectal cancer (CRC) COLO320DM and DLD-1 cells were treated with 10 μM of each TI compound for 24 h. (**A**) mRNA expression levels of the indicated β-catenin target genes (*AXIN2*, *BIRC5*, *CCND1*, *cMYC*, and *FGF20*) were quantified using quantitative polymerase chain reaction (qPCR). XAV939 was used as a reference control. Data represent the mean ± standard deviation (SD) of three independent experiments. * *p* < 0.05, ** *p* < 0.01, *** *p* < 0.001 versus respective DMSO-treated cells. (**B**) Whole cell lysates were subjected to immunoblotting for detection of active β-catenin (ABC), total β-catenin, TNKS1/2, and AXIN2. β-actin was used as a loading control. The density of each band was measured by Image J software and normalized to that of β-actin. Data represent the mean ± SD of three independent experiments. * *p* < 0.05, ** *p* < 0.01 versus respective DMSO-treated cells. (**C**) COLO320DM and DLD-1 cells were treated with 10 μM TI-12403 and immunostained for AXIN2 (green) and β-catenin (red). Nuclear DNA was counterstained with DAPI (blue). Scale bar, 20 μm. (**D**) Quantification of the mean fluorescence intensity in Figure 1C. * *p* < 0.05, ** *p* < 0.01, *** *p* < 0.001 versus corresponding cells. (**E**) COLO320DM and DLD-1 cells were transfected with either TOP- (wild-type TCF binding sites) or FOP- (mutated TCF binding sites) flash reporter plasmid and then treated with 10 μM TI-12403 for 48 h. Transcriptional activity was measured with a luciferase reporter assay system and was calculated by dividing the TOP ratio by the FOP ratio (TOP/FOP ratio). Data represent the mean ± SD of three independent experiments. *** *p* < 0.001 versus respective DMSO-treated cells.

We next determined whether the eight compounds inhibited β-catenin signaling in APC-mutated CRC cells. COLO320DM cells were treated with 10 μM of each compound for 24 h. Among the compounds, N-([1,2,4]triazolo[4,3-a]pyridin-3-yl)-1-(2-cyanophenyl)piperidine-4-carboxamide (TI-12403) markedly downregulated mRNA levels of β-catenin target genes in COLO320DM cells (Figure 1A and Supplementary Figure S2). XAV939 is the first potent inhibitor of TNKS1/2 and was used in this study as a reference control. TI-12403 also downregulated mRNA levels of β-catenin target genes in human CRC DLD-1 cells harboring the APC mutation (Figure 1A). We confirmed β-catenin and AXIN2 protein levels in COLO320DM and DLD-1 cells using Western blotting. TI-12403 induced AXIN2 and TNKS1/2 accumulation and inhibited the active- β-catenin (ABC) protein (Figure 1B). Immunofluorescence staining confirmed the reduction of ABC and accumulation of AXIN2 in both cell lines (Figure 1C,D). To determine whether TI-12403 regulates β-catenin/TCF-dependent transcriptional activity, we used a luciferase reporter assay system with TOPFlash (a wild-type TCF binding site) or FOPFlash (a mutated TCF binding site) in COLO320DM and DLD-1 cells. Results showed that TI-12403 suppressed β-catenin-dependent reporter activity in both cell lines to a greater extent than that by XAV939 (Figure 1E). To determine whether TI-12403 inhibits PARP-1 in addition to TNKS, PARP-1 activity was measured using a cell-free PARP-1 enzyme assay system. TI-12403 at 10 μM showed 7% inhibitory activity of PARP-1, (Supplementary Table S2). These data suggest that TI-12403, a specific TNKS inhibitor, specifically suppresses β-catenin signaling in human CRC cells.

**Figure 2:**
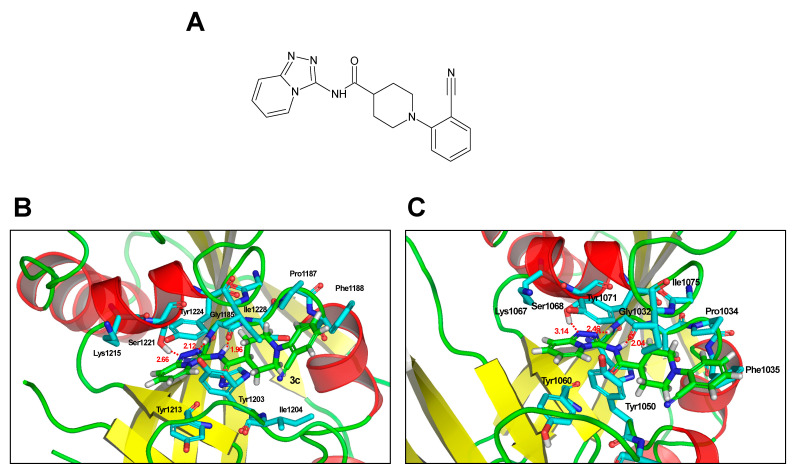
Predicted docking pose of TI-12403 in TNKS1 and TNKS2. (**A**) Chemical structure of TI-12403. (**B**,**C**) The analysis was performed by aligning the triazol ring atoms between TI-12403 and X-ray ligand (TNKS1; 4KRS.pdb, TNKS2; 3P0Q.pdb). Hydrogen bonds with relevant active site residues are shown as red dashes.

Several TNKS inhibitors have a triazolopyridazine functional group or a dihydrothiazolotriazole group with an X-ray crystal structure [23,24]. We superimposed the released TNKS1 X-ray crystal structure (PDB code: 4KRS) with the dihydrothiazolotriazole complex structure for TI-12403 (Figure 2A,B). The Ser1221 side chain of hydroxyl forms a hydrogen bond with triazololpyridine, and the Gly1185 backbone chain oxygen forms a hydrogen bond with the triazololpyridyl group. The amide linker of TI-12403 also forms a hydrogen bond with the carbonyl oxygen of Gly1185. Additional hydrophobic interactions exist between the cyanophenyl ring of TI-12403 and the binding pocket involving Pro1187, Phe1188, and Ile1204 of TNKS1. In case of the docking position between TI-12403 and TNKS2 (PDB code: 3P0Q), the amide linker of TI-12403 forms two hydrogen bonds with carbonyl oxygen and NH hydrogen of Gly1032 (Figure 2C). However, the Ser1068 side chain of hydroxyl does not form a hydrogen bond with triazolopyridine at the intermolecular distance 3.14 Å. Our docking studies suggested that TI-12403 bound to both the nicotinamide pockets of TNKS1 and TNKS2. The IC_50_ value of TNKS1 and TNKS2 is shown in Table 1.

### 2.2. TI-12403 Inhibits Human CRC Cell Growth In Vitro and In Vivo

**Figure 3:**
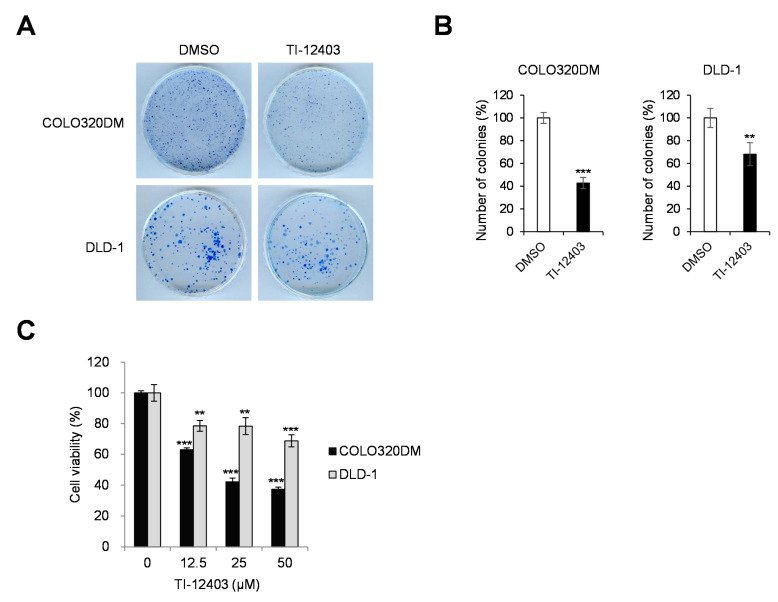
TI-12403 inhibits the growth of adenomatous polyposis coli (APC)-mutated colorectal cancer (CRC) cells. (**A**) COLO320DM and DLD-1 cells were treated with 10 μM TI-12403 and then incubated for 14 days. Colonies with more than 50 cells were counted. (**B**) Colony formation assay results presented as bar graphs showing the number of colonies. The bar graph represents the normalized values to control group. *** *p* < 0.001, ** *p* <0.01 compared to vehicle controls. (**C**) COLO320DM and DLD-1 cells were incubated with TI-12403 at the indicated concentrations. Cell viability was determined by MTT assay 72 h after treatment. ** *p* < 0.01, *** *p* < 0.001 compared to respective DMSO-treated cells.

We assessed whether TI-12403-mediated inhibition of Wnt/β-catenin signaling affects CRC cell proliferation. COLO320DM and DLD-1 cells were treated with TI-12403, and growth inhibition was determined using a colony formation assay. TI-12403 inhibited the viability of COLO320DM and DLD-1 cells (Figure 3A,B). Consistent with these findings, TI-12403 treatment significantly inhibited the viability of COLO320DM and DLD-1 cells, as determined by MTT assay (Figure 3C).

**Figure 4:**
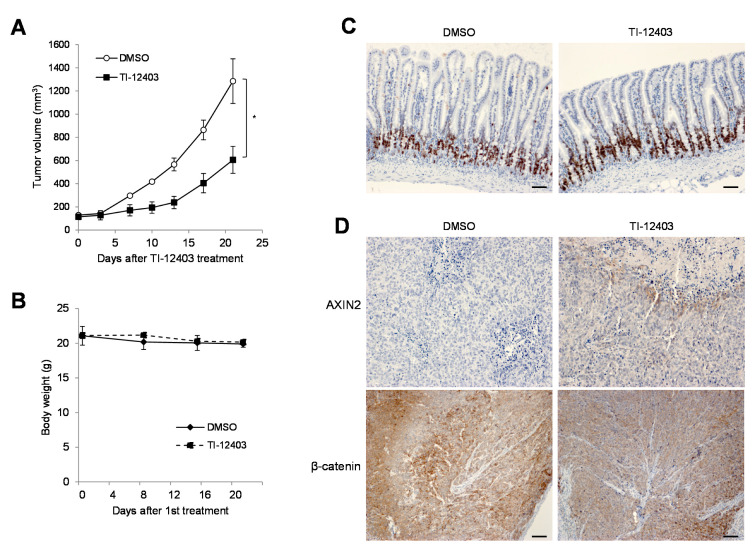
TI-12403 suppressed the growth of DLD-1 xenograft tumors in nude mice. (**A**) DLD-1 cells were subcutaneously injected into the thigh of the right hind leg of BALB/c nu/nu mice (n = 4 per group). One week after tumor cell injection, TI-12403 (20 mg/kg) or DMSO (control) was intraperitoneally administered once per day for 14 days. The longest (L) and shortest (W) tumor axes were measured, and tumor volume (mm^3^) was calculated as L ×W^2^/2. The data shown represent the average tumor volume (* *p* < 0.05). (**B**) Body weights of DLD-1 tumor xenograft mice were determined once weekly during the experiments. (**C**,**D**) Xenograft tumors and small intestines in each group were harvested and extracted completely after 21 days. (**C**) Small intestine sections from TI-12403- or DMSO-treated DLD-1 xenograft mice were immunohistochemically stained with the cell proliferation marker Ki67 (brown). Scale bar, 100 μm. (**D**) Immunohistochemical staining of β-catenin and AXIN2 (brown) in tumor tissues. Scale bar, 100 μm.

We next determined whether the in vitro antitumor effect of TI-12403 could be translated to an in vivo xenograft mouse model. BALB/c-nu/nu mice were subcutaneously implanted with DLD-1 cells in the right hind leg. When the tumors were palpable (average diameter approximately 100 mm^3^; 7 days post-implantation), mice were intraperitoneally administered with 20 mg/kg TI-12403 or DMSO (control vehicle) once per day for 14 days. Compared to the vehicle, TI-12403 inhibited DLD-1 cell-derived tumor growth by 53% (Figure 4A). We did not observe any differences in body weights between control mice and TI-12403-treated mice (Figure 4B). Therefore, these results suggested that TI-12403 exhibited antitumor activity in APC-mutated CRCs both in vitro and in vivo.

### 2.3. TI-12403 Decreases β-Catenin Levels and Increases AXIN2 Levels in DLD-1 Xenograft Mouse Tumors without Inducing Intestinal Toxicity

Wnt/β-catenin signaling plays an important role in maintaining normal tissue homeostasis in the intestine [25,26]. Therefore, high doses of TNKS inhibitors induce intestinal toxicity [19,20]. To determine TI-12403 toxicity, we examined the small intestine of mice treated with TI-12403 for 21 days in DLD-1 xenograft tumor efficacy study. The small intestine tissue was embedded in formalin-fixed paraffin (FFPE) and stained with the proliferation marker Ki67. Ki67 expression in the small intestine of TI-12403-treated mice was similar to that observed in the control mice (Figure 4C). Additionally, the TI-12403-treated small intestine did not appear to have damaged crypts or villi, and the small intestine was similar to that observed in the control group. Twenty-one days after TI-12403 treatment, we analyzed β-catenin and AXIN2 levels in tumor tissues of DLD-1 xenograft mice by immunohistochemistry assay. TI-12403-treated tumors had significantly increased AXIN2 protein expression levels and inhibited β-catenin levels compared to that in the controls (Figure 4D), suggesting that TI-12403 exhibited antitumor activity and did not have intestinal toxicity in vivo.

### 2.4. Combination Treatment with TI-12403 and 5-FU Synergistically Inhibited Human CRC Cell Growth

**Figure 5:**
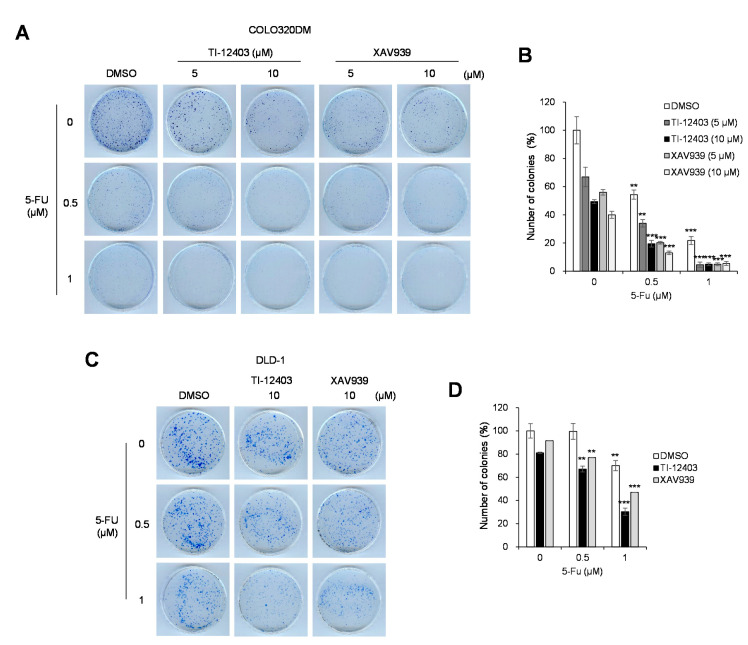
TI-12403 enhances chemosensitivity in COLO320DM and DLD-1 cells. (**A**,**B**) COLO320DM and (**C**,**D**) DLD-1 cells were treated with the indicated dose of TI-12403 for 2 h before treatment with the indicated doses of 5-fluorouracil (5-FU). Colonies were counted 10 days after 5-FU treatment. Colony numbers are normalized to the average value of the control growth values for each cell line. Colonies consisting of more than 50 cells were scored as survival colonies. Colony formation assay results are presented as bar graphs showing the number of colonies. The bar graph represents the normalized values to control group. ** *p* <0.01, *** *p* < 0.001 compared to each drug alone.

5-fluorouracil (5-FU) is commonly used in chemotherapy for patients with advanced CRC [26,27]. We evaluated whether treatment with a combination of 5-FU and TI-12403 produced synergistic effects. COLO320DM and DLD-1 cells were treated with the indicated concentrations of TI-12403 and 5-FU, and cell viability was assessed using a colony formation assay. Compared to TI-12403 or 5-FU treatment alone, combination treatment showed a stronger synergistic effect than XAV939 in COLO320DM (Figure 5A,B) and DLD-1 cells (Figure 5C,D). These results indicated that TI-12403 and 5-FU combination treatment synergistically inhibited COLO320DM and DLD-1 cell growth.

## 3. Discussion

Although dysregulated Wnt/β-catenin signaling is one of the distinguishing features of CRC, efforts continue to be carried out to identify targets that inhibit this pathway for clinical use. TNKS, which degrades AXIN in the β-catenin destruction complex, has been suggested as an attractive target for cancer therapy [5]. Here, we identified two commercial compounds through virtual screening, based on which we designed and synthesized 17 compounds. We discovered that TI-12403, a small-molecule compound, was the most potent among the compounds identified by in vitro screening and strongly inhibited TNKS1/2 but not PARP-1 activity. TI-12403 stabilized AXIN2, reduced active β-catenin, and showed anticancer effects in human APC-mutant CRC cells, thus making it a potential TNKS1 inhibitor.

Tanaka et al. reported that the APC mutation length predicts the sensitivity of CRC cells to TNKS inhibitors [21]. COLO320DM cells have an APC mutation that lacks all 20-amino acid repeats (20-AAR), which are highly dependent on β-catenin signaling and sensitive to TNKS inhibitors. DLD-1 cells are relatively less sensitive compared to COLO320DM cells to TNKS inhibitors, despite the cells having mutant APCs with two 20-AARs. Consistent with this report, we observed that TI-14203 reduced COLO320DM cell viability more effectively compared to DLD-1 cells (Figure 3). Although TNKS inhibitors reduce APC mutation-dependent cell viability, it is worthy to mention that TI-12403 inhibit proliferation of DLD-1 cells. TNKS is involved in other oncoproteins networks, including Hippo-Yes associated protein (YAP)/TAZ (PDZ-binding motif) signaling pathway. Inhibition of TNKS stabilizes the AMOT family of proteins by inhibiting RNF146 axis-mediated degradation, thereby inhibiting YAP oncogenic function [25]. Notably, TI-12403 inhibited YAP target gene expression in DLD-1 cells. However, we did not observe the expression of YAP target genes in COLO320DM cells with either TI-12403 or DMSO treatment (Supplementary Figure S3). Since E-cadherin can positively regulate YAP signaling, E-cadherin-deficient COLO320DM cells did not appear to express the YAP target genes [28]. We speculated that the anticancer effects of TI-12403 on DLD-1 cells might be due to TI-12403-mediated inhibition of YAP signaling. Therefore, TI-12403 is expected to have a therapeutic effect in a wider variety of cancers where YAP signaling is upregulated.

A few selective TNKS inhibitors are being developed; however, most of their evaluation remains preclinical. Since β-catenin is a key in maintaining intestinal homeostasis, TNKS inhibitors such as G007-LK, XAV939, and G-631 have side effects leading to intestinal toxicity or severe weight loss in mice [19,20,29]. TI-12403 showed reduced intestinal toxicity or body weight change (Figure 4). Additionally, TI-12403 had excellent metabolic stability in human liver microsomal and plasma and did not show cytochrome P450 inhibitory activity (Supplementary Table S3). Altogether, TI-12403 exerted no significant toxicity due to its high metabolic stability in mice. We postulate that TI-12403 requires further evaluation for effective drug development but has potential as a therapeutic agent against cancer.

The current first-line therapeutic agent used clinically in CRC is 5-FU and it has improved the overall survival rate of patients with CRC; however, its clinical use is limited due to its toxicity and chemoresistance. Combination treatment is an effective clinical strategy for anticancer therapy in CRC [26,30]. Previous studies have reported that APC mutations contribute to 5-FU resistance in CRC cells [29,31]. Recently, it has been suggested that TNKS inhibitors reduced 5-FU resistance in APC mutant cells [29]. Consistent with the aforementioned report, we found that combination treatment with TI-12403 and 5-FU significantly inhibited COLO32DM and DLD-1 cell viability (Figure 5). Thus, TNKS inhibitors can be considered as therapeutic agents for combination treatment in CRC.

In summary, TI-12403 exhibited potent TNKS inhibitor activity and cytotoxicity toward CRC cells. TI-12403 induced AXIN2 expression and downregulated β-catenin, increasing the sensitivity of cancer cells. Moreover, TI-12403 and 5-FU combination treatment considerably inhibited cell proliferation. Therefore, the novel TNKS inhibitor TI-12403 may be effective in the treatment of APC-mutant CRC and could have further potential as an adjuvant when used in combination with 5-FU.

## 4. Materials and Methods

### 4.1. Chemical Synthesis

All derivatives (3a-q, 5a-b) were synthesized by performing amide coupling reactions with commercially available starting materials (Enamine, Monmouth Jct., NJ, USA), including [1,2,4]triazolo[4,3-a]pyridin-3-amine (1; Supplementary Scheme S1), 7-methyl-[1,2,4]triazolo[4,3-a]pyridin-3-amine (4a), or 5,6,7,8-tetrahydro-[1,2,4]triazolo[4,3-a]pyridin-3-amine (4b; Supplementary Scheme S2). Proton nuclear magnetic resonance spectra of all chemicals were recorded and are provided in the Supplementary Materials and Methods section.

### 4.2. In Vitro Enzyme Assay

TNKS1 and TNKS2 activities of the compounds were measured using colorimetric activity assays (BPS Bioscience, San Diego, CA, USA) according to the manufacturer’s protocol, and their IC_50_ values were determined based on the TNKS1 and TNKS2 activities.

### 4.3. Cell Culture

Human CRC cells (COLO320DM and DLD-1 cells) were purchased from American Type Culture Collection (ATCC, Manassas, VA, USA). The cells were authenticated at Cosmogenetech (Seoul, Korea) based on the ATCC cellular information and cultured in Roswell Park Memorial Institute (RPMI) 1640 medium (Welgene, Gyeongsangbuk-do, Korea) or Dulbecco’s modified Eagle’s medium (DMEM, Welgene) supplemented with 10% fetal bovine serum (FBS) (Welgene) and 100 units/mL penicillin–streptomycin (Gibco, Grand Island, NY, USA), followed by incubation at 37 °C in a 5% CO_2_ incubator.

### 4.4. Cell Proliferation Assay

Cells were seed in 96-well plates at a density of 1 × 10^3^ cells/well in triplicate, treated with 10 μM TI-12304 or XAV939 for 72 h. Cell proliferation was evaluated by 3-(4,5-dimethylthiazol-2-yl)-2,5 diphenyl tetrazolium bromide (MTT) assay (Sigma-Aldrich) according to the manufacturer’s recommendations. Briefly, 10 µL of MTT (0.5 mg/mL) was added to the culture medium and incubated for 2 h, and the absorbance at 540 nm was determined by a Multiskan EX plate reader (Thermo LabSystems, Waltham, MA, USA). For colony formation assay, cells were seeded at 500 cells per 60 mm dish and incubated at 37 °C and 5% CO_2_. After 24 h, the cells were treated with 10 μM TI-12403 or XAV939. In the combination treatment experiment, cells were treated with the indicated dose of TI-12403 for 2 h before treatment with the indicated doses of 5-fluorouracil (5-FU). After 10 days, the colonies were fixed and stained with 1.5% methylene blue (Sigma Aldrich) in methanol solution for visualization. Colonies containing >50 cells were counted.

### 4.5. Immunoblot Analysis

Cell lysates were prepared by extracting proteins with TNN buffer (40 mM Tris-Cl pH 8.0, 0.2% NP-40, 120 mM NaCl) supplemented with a protease inhibitor cocktail (Thermo Fisher Scientific, Rockford, IL, USA). Western blot analysis was performed as previously described [32]. Details of the primary antibodies used in this study are provided in the Supplementary Materials and Methods section.

### 4.6. RNA Extraction and Quantitative Polymerase Chain Reaction (qPCR) Analysis

RNA was extracted using TRIzol^®^ RNA isolation reagent (Thermo Fisher Scientific). Reverse transcription of RNA to cDNA was performed using AccuPower^®^ CycleScript™ RT PreMix (Bioneer, Daejeon, Korea). The cDNA was quantified using real-time PCR with SYBR Green/fluorescein qPCR master mix (Thermo Scientific, Carlsbad, CA, USA) on a Lightcycler 96 system (Roche Diagnostics, Mannheim, Germany) according to the manufacturer’s protocol. The sequences of the primers used are provided in the Supplementary Materials and Methods section.

### 4.7. Luciferase Assay

Cells were transfected with TOPFlash or FOPFlash using Lipofectamine 2000 (Invitrogen, Carlsbad, CA, USA). After 24 h, transfected cells were treated with TI-12403, XAV939, or DMSO for 48 h. For TOP/FOP-Flash reporter assays, cells were lysed using cell lysis buffer (Promega, Madison, WI, USA), and then luciferase activity was measured using the Luciferase Reporter Assay System (Promega) on a microplate luminometer (Victor ×2; Perkin Elmer, Waltham, MA, USA).

### 4.8. Immunofluorescence Staining

COLO320DM and DLD-1 cells were fixed in 4% paraformaldehyde for 15 min at 25 °C and permeabilized with 0.1% Triton X-100 in PBS for 20 min. The cells were then incubated with a 1:100 dilution of anti-anti-active β-catenin (ABC) and Axin2 antibody overnight at 4 °C. Next, the cells were incubated with Alexa Fluor 488-conjugated anti-mouse IgG antibody (Abcam, Cambridge, UK) and Alexa Fluor 594-conjugated anti-rabbit IgG antibody at a 1:400 dilution for 1 h at room temperature. The slides were mounted in mounting medium (DAKO, Santa Clara, CA, USA) with DAPI (Thermo Fisher Scientific) before imaging. Images were acquired using an LSM880 laser scanning microscope (ZEISS, Jena, Germany). Fluorescence images were captured using appropriate filters. Images were analyzed using ImageJ and ZEN software.

### 4.9. Tumor Xenograft Mouse Models

All animal experiments were reviewed and approved by the Institutional Animal Care and Use Committee of the Korea Institute of Radiological and Medical Sciences (Kirams2018-0063). DLD-1 cells (2 × 10^6^) were implanted subcutaneously into the thigh of the right hind leg of six-week-old mice. When tumor volumes reached approximately 100 mm^3^, TI-12403 (20 mg/kg) was administered intraperitoneally once per day for 14 days. The body weights of the mice were measured once a week.

### 4.10. Immunohistochemistry (IHC)

Mice treated with DMSO or TI-12403 were sacrificed, and the intestines and tumors were dissected. The tissues were fixed in 4% paraformaldehyde (in PBS solution) overnight and embedded in paraffin. After sectioning, the intestines were stained with Ki67. The tumor tissues were stained with an anti-β-catenin antibody and anti-Axin2 antibody.

### 4.11. Statistical Analysis

Results are shown as the mean ± standard deviation (SD). Data were analyzed using two-tailed Student’s *t*-tests. Differences between groups with *p*-values < 0.05 were considered statistically significant.

## Figures and Tables

**Table 1:** IC_50_ (μM) of TI-12403 against TNKS1 and TNKS2.

Compounds	TNKS1	TNKS2
TI-12403	0.029	0.060

## Data Availability

Not applicable.

## Supplementary Materials

The following are available online at https://www.mdpi.com/article/10.3390/ijms22147330/s1.
