# Supplementary material for: Discovery of a Novel Triazolopyridine Derivative as a Tankyrase Inhibitor
Source: Int J Mol Sci. 2021 Jul 8;22(14):7330. doi: 10.3390/ijms22147330 (PMC8303674; doi:10.3390/ijms22147330)
Supplement: Supplementary file 1 [file ijms-22-07330-s001.zip › ijms-1280356-supplementary.pdf]

Supplementary materials

Proton nuclear magnetic resonance (NMR) analysis

^{1}H NMR spectra were recorded using an Avance-500 spectrometer (Bruker Daltonics Inc., Billerica, MA, USA) at 500 MHz. Chemical shifts are reported in ppm, and Me4Si was used as the reference standard. Mass spectra were recorded using a JMS-700 MStation (EI mode; JEOL Ltd., Tokyo, Japan). Chemical shifts are expressed as δ values relative to trimethylsilyl as the internal standard, J values are expressed in Hz, and spectra were recorded in $CDCl_3$ or hexadeutero-DMSO [(DMSO-*d*$_6$); Sigma-Aldrich, St. Louis, MO, USA]. Multiplicities of signals are indicated by the following symbols: s (singlet), d (doublet), t (triplet), m (multiplet), and brs (broad singlet). Reaction products were purified by flash column chromatography using silica gel 60 (230–400 mesh) and monitored by thin-layer chromatography with precoated silica gel 60 F254 (Merck, Darmstadt, Germany). Spots were visualized under UV light (254 nm) after staining with PMA or Hanessian solution.

N-([1,2,4]triazolo[4,3-a]pyridin-3-yl)-1-phenylpiperidine-4-carboxamide (TI-12401)

^{1}H NMR ($CDCl_3$-*d*, 400 MHz): δ 11.65 (1H, s), 7.91 (1H, d, J = 4.0Hz), 7.67 (1H, d, J = 8.0Hz), 7.27 (3H, m), 6.97 (2H, d, J = 8.0Hz), 6.9 (2H, m), 3.76 (2H, dt, J=11.0 and 3.1Hz), 2.9 (3H, td, J=10.0 and 3.2Hz), 2.19 (2H, dd, J=14.0 and 2.0Hz), 2.07(2H, ddd, J=24.0, 12.0 and 4.0Hz)

N-([1,2,4]triazolo[4,3-a]pyridin-3-yl)-1-(4-nitrophenyl)piperidine-4-carboxamide (TI-12402)

^{1}H NMR (DMSO-*d*$_6$, 400 MHz): δ 10.96 (1H, s), 8.06 (2H, d, J = 8.0Hz), 7.93 (1H, d, J = 4.0Hz), 7.74 (1H, d, J = 8.0Hz), 7.39 (1H, ddd, J = 8.4, 6.6 and 1.8Hz), 7.07 (2H, d, J = 8.0Hz), 6.97 (1H, t, J = 6.0Hz), 4.13 (2H, d, J = 12.0Hz), 3.14 (2H, t, J = 12.0Hz), 2.89 (1H, m), 2.05 (2H, d, J = 12.0Hz), 1.75 (2H, m)

N-([1,2,4]triazolo[4,3-a]pyridin-3-yl)-1-(2-cyanophenyl)piperidine-4-carboxamide (TI-12403)

^{1}H NMR (DMSO-*d*$_6$, 400 MHz): δ 10.96 (1H, s), 7.96 (1H, d, J = 8.0Hz), 7.73 (1H, m), 7.61 (1H, t, J = 8.0Hz), 7.4 (1H, ddd, J = 12.0, 4.0 and 0.8Hz), 7.22 (1H, d, J = 8.0Hz), 7.11 (1H, t, J = 8.0Hz), 6.99 (1H, t, J= 6.0Hz), 3.58 (2H, d, J=12.0Hz), 2.91 (2H, t, J = 12.0Hz), 2.50 (1H, m) 2.10 (2H, d, J = 12.0Hz), 1.91 (2H, t, J = 6.0Hz)

N-([1,2,4]triazolo[4,3-a]pyridin-3-yl)-1-(2-nitrophenyl)piperidine-4-carboxamide (TI-12404)

^{1}H NMR (DMSO-*d*$_6$, 400 MHz): δ 10.95 (1H, s), 7.95 (1H, d, J = 8.0Hz), 7.81 (1H, dd, J = 8.0 and 4.0Hz), 7.75 (1H, d, J = 8.0Hz), 7.59 (1H, td, J = 8.0, and 4.0Hz), 7.38 (2H, m), 7.13 (1H, t, J = 8.0Hz), 7.00 (1H, t, J = 4.0Hz), 3.27 (2H, d, J = 12.0Hz), 2.91 (2H, t, J = 12.0Hz), 2.71 (1H, m), 2.03 (2H, d, J = 12.0Hz), 1.85 (2H, m)

N-([1,2,4]triazolo[4,3-a]pyridin-3-yl)-1-(2-nitro-4-(trifluoromethyl)phenyl)piperidine-4-carboxamide (TI-12405)

^{1}H NMR (DMSO-*d*$_6$, 400 MHz): δ 10.97 (1H, s), 8.16 (1H, d, J = 4.0Hz), 7.87 (1H, d, J = 4.0Hz), 7.85 (1H, d, J = 2.0Hz), 7.75 (1H, dt, J = 10.6 and 0.4Hz), 7.49 (1H, d, J = 8.0Hz), 7.40 (1H, ddd, J = 10.2, 6.0 and 1.4Hz), 6.99 (1H, td, J = 7.5, 7.5 and 3.2Hz), 3.43 (2H, d, J = 16.0Hz), 3.07 (2H, t, J = 12.0Hz), 2.79 (1H, m), 2.05 (2H, d, J = 12.0Hz), 1.85 (2H, t, J = 12.0Hz)

N-([1,2,4]triazolo[4,3-a]pyridin-3-yl)-1-(4-(trifluoromethyl)phenyl)piperidine-4-carboxamide (TI-12406)

^{1}H NMR (DMSO-*d*$_6$, 400 MHz): δ 10.94 (1H, s), 7.93 (1H, d, J = 8.0Hz), 7.74 (1H, d, J = 12.0Hz), 7.50 (2H, d, J = 12.0Hz), 7.39 (1H, t, J = 8.0Hz), 7.10 (2H, d, J = 8.0Hz), 6.97 (1H, t, J = 6.0Hz), 3.97 (2H, d, J = 16.0Hz), 2.95 (2H, t, J = 12.0Hz), 2.81 (1H, m), 2.02 (2H, d, J = 12.0Hz), 1.76 (2H, m)

N-([1,2,4]triazolo[4,3-a]pyridin-3-yl)-1-(4-fluoro-2-nitrophenyl)piperidine-4-carboxamide (TI-12407)

^{1}H NMR (DMSO-d_6, 400 MHz): δ 10.94 (1H, s), 7.94 (1H, d, J = 4.0Hz), 7.83 (1H, dd, J = 8.0 and 4.0Hz), 7.75 (1H, d, J = 8.0Hz), 7.52 (2H, m), 7.40 (1H, dd, J = 12.0 and 6.0Hz), 6.99 (1H, t, J = 8.0Hz), 3.20 (2H, d, J = 12.0Hz), 2.88 (2H, t, J = 12.0Hz), 2.69 (1H, m), 2.03 (2H, d, J = 8.0Hz), 1.82 (2H, m)

N-([1,2,4]triazolo[4,3-a]pyridin-3-yl)-1-(4-acetylphenyl)piperidine-4-carboxamide (TI-12408)

^{1}H NMR (DMSO-d_6, 400 MHz): δ 10.95 (1H, s), 7.93 (1H, d, J = 8.0Hz), 7.81 (2H, d, J = 8.0Hz), 7.74 (1H, d, J = 12.0Hz), 7.39 (1H, ddd, J = 8.4, 6.4 and 1.6Hz), 6.99 (3H, m), 4.04 (2H, d, J = 12.0Hz), 3.00 (2H, t, J = 12.0Hz), 2.84 (1H, m), 2.45 (3H, s), 2.02 (2H, d, J = 12.0Hz), 1.74 (2H, m)

N-([1,2,4]triazolo[4,3-a]pyridin-3-yl)-1-(4-chloro-2-nitrophenyl)piperidine-4-carboxamide (TI-12409)

^{1}H NMR (DMSO-d_6, 400 MHz): δ 10.95 (1H, s), 7.95 (2H, m), 7.75 (1H, d, J = 8.0Hz), 7.65 (1H, dd, J = 12.0 and 8.0Hz), 7.39 (2H, m), 6.99 (1H, t, J = 8.0Hz), 3.26 (2H, d, J = 12.0Hz), 2.92 (2H, t, J = 12.0Hz), 2.72 (1H, m), 2.03 (2H, d, J = 8.0Hz), 1.83 (2H, m)

N-([1,2,4]triazolo[4,3-a]pyridin-3-yl)-1-(4-cyanophenyl)piperidine-4-carboxamide (TI-12410)

^{1}H NMR (DMSO-d_6, 400MHz): δ 10,94(1H, s), 7.93(1H, d, J=8.0Hz), 7.74(1H, d, J=8.0Hz), 7.58(1H, d, J=8.0Hz), 7.39(1H, dd, J=8.0 and 8.0Hz), 7.06(2H, d, J=8.0Hz), 6.97(1H, m), 4.03(2H, d, J=12.0Hz), 3.01(2H, m), 2.83(1H, m), 2.02(2H, d, J=16Hz), 1.73(2H, m)

N-([1,2,4]triazolo[4,3-a]pyridin-3-yl)-1-(5-nitropyridin-2-yl)piperidine-4-carboxamide (TI-12411)

^{1}H NMR (DMSO-d_6, 400MHz): δ 10,97(1H, s), 8.98(1H, d, J=2.0Hz), 8.23(1H, dd, J=2.0 and 4.0Hz), 7.94(1H, d, J=8.0Hz), 7.74(1H, d, J=8.0Hz), 7.39(1H, dd, J=2.0 and 8.0Hz), 6.99(2H, m), 4.59(2H, d, J=12.0Hz), 3.22(2H, m), 2.94(1H, m), 2.08(2H, d, J=12.0Hz), 1.70(2H, m)

N-([1,2,4]triazolo[4,3-a]pyridin-3-yl)-1-(2-cyano-4-nitrophenyl)piperidine-4-carboxamide (TI-12412)

^{1}H NMR (DMSO-d_6, 400 MHz): δ 11.00 (1H, s), 8.54 (1H, d, J = 4.0Hz), 8.3 (1H, dd, J = 10.0 and 2.0Hz), 7.96 (1H, d, J=4.0Hz), 7.75 (1H, d, J = 8.0Hz), 7.39 (1H, ddd, J=6.6, 4.0 and 1.4Hz), 7.30 (1H, d, J = 12.0Hz), 6.98 (1H, t, J= 6.0Hz), 4.05 (2H, d, J=12.0Hz), 3.25 (2H, t, J = 12.0Hz), 2.87 (1H, m), 2.13 (2H, d, J = 12.0Hz), 1.885 (2H, d, J = 12.0Hz)

N-([1,2,4]triazolo[4,3-a]pyridin-3-yl)-1-benzylpiperidine-4-carboxamide (TI-12413)

^{1}H NMR (MeOD-d_4, 400 MHz): δ 7.97(d, 1H, *J*=8.0Hz), 7.69(d, 1H, *J*=8.0Hz), 7.47(m, 1H), 7.32(m, 5H), 7.03(m, 1H), 3.62(s, 2H), 3.05(m. 2H), 2.63(m, 1H), 2.23(m, 2H), 2.01(m, 2H), 1.90(m, 2H)

N-([1,2,4]triazolo[4,3-a]pyridin-3-yl)-1-benzoylpiperidine-4-carboxamide (TI-12414)

^{1}H NMR (DMSO-d_6, 400 MHz): δ 7.94(d, 1H, *J*=8.0Hz), 7.75(m, 1H), 7.45(m, 3H), 7.40(m, 4H), 6.98(m, 1H), 4.51(bs, 1H), 3.66(bs. 1H), 3.15(bs, 1H), 2.94(bs, 1H), 2.84(m, 1H), 2.05(m, 2H), 1.69(m, 2H)

N-([1,2,4]triazolo[4,3-a]pyridin-3-yl)-1-(4-(N,N-diethylsulfamoyl)-2-methylphenyl)piperidine-4-carboxamide (TI-12415)

^{1}H NMR (MeOD-d_4, 400 MHz): δ 8.15(d, 1H, *J*=4.0Hz), 8.02(m, 1H), 7.87(m, 1H), 7.75(m, 1H), 7.50(m, 3H), 7.41(d, 1H, *J*=8.0Hz), 7.04(d, 1H, *J*=8.0Hz), 3.72(m, 1H), 3.53(m. 2H), 3.31(m, 4H), 3.16(m, 2H), 2.10(m, 2H), 2.02(m, 2H), 1.87(m, 1H), 1.15(m, 6H)

N-(7-methyl-[1,2,4]triazolo[4,3-a]pyridin-3-yl)-1-(2-nitrophenyl)piperidine-4-carboxamide (TI-12416)

^{1}H NMR (DMSO-d_6, 400 MHz): δ 8.13(d, 1H, *J*=8.0Hz), 7.80(dd, 1H, *J*=8.0Hz and 2.0Hz), 7.57(m, 1H), 7.34(m, 2H), 7.12(d, 1H, *J*=8.0Hz), 6.84(dd, 1H, *J*=8.0Hz and 2.0Hz), 3.24(m. 2H), 2.88(m, 2H), 2.58(m, 1H), 2.38(bs, 3H), 2.03(m, 2H), 1.76(m, 2H)

1-(2-nitrophenyl)-N-(5,6,7,8-tetrahydro-[1,2,4]triazolo[4,3-a]pyridin-3-yl)piperidine-4-carboxamide (TI-12417)

^{1}H NMR (DMSO-d_6, 400 MHz): δ 7.79(dd, 1H, *J*=8.0Hz and 2.0Hz), 7.58(m, 1H), 7.31(d, 1H, *J*=8.0Hz), 7.10(m, 1H), 3.70(m, 2H), 3.20(m. 2H), 2.90(m, 2H), 2.78(m, 2H), 1.94(m, 4H), 1.84(m, 2H), 1.70(m, 2H)

Antibodies for immunoblot analysis

The primary antibodies used were anti-active β-catenin (ABC, 05-665; Millipore, Billerica, MA, USA), anti-β-catenin (610154; BD Transduction Laboratories™, Franklin Lakes, NJ), anti-Axin2 (76G6; Cell Signaling Technology), anti-Yes-associated protein/WW Domain Containing Transcription Regulator 1 (YAP/TAZ, #8418; cell signaling), Cysteine-rich angiogenic inducer 61 (CYR61, sc-374129; Santa Cruz Biotechnology), connective tissue growth factor (CTGF, sc-73069; Santa Cruz Biotechnology).anti-glyceraldehyde 3-phosphate dehydrogenase (GAPDH, sc-47724; Santa Cruz Biotechnology), and anti-β-actin (A5441; Sigma-Aldrich).

Primer Sequences for quantitative polymerase chain reaction (qPCR)

The sequences of the primers targeting human *AXIN2*, *CCND1*, *BIRC5*, *cMYC*, *FGF20*, *AMOTL-2*, *CYR61*, *CTGF*, and *GAPDH* are as follows: *AXIN2*, 5′-TACACTCCTTATTGGGCGATC-3′ (forward) and 5′-TTGGCTACTCGTAAAGTTTTGGT-3′ (reverse); *CCND1*, 5′-TCTACACCGACAACTCCATCCG-3′ (forward) and 5′-TCTGGCATTTTGGAGAGGAAGTG-3′ (reverse); *BIRC5*, 5′-CCAGATGACGACCCCATAGAG-3′ (forward) and 5′-TTGTTGGTTTCCTTTGCAATTTT-3′ (reverse); *cMYC*, 5′-CCTGGTGCTCCATGAGGAGAC-3′ (forward) and 5′-CAGACTCTGACCTTTTGCCAGG-3′ (reverse); *FGF20*, 5′-TAGAGGTGTGGACAGTGGTCTC-3′ (forward) and 5′-CTTCAAACTGCTCCCTAAAGATGC-3′ (reverse); *AMOTL-2* 5'-AGTGAGCGACAAACAGCAGACG-3' (forward) and 5'-ATCTCTGCTCCCGTGTTTGGCA-3' (reverse); *CYR61*, 5'-GGAAAAGGCAGCTCACTGAAGC-3' (forward) and 5'-GGAGATACCAGTTCCACAGGTC-3' (reverse); *CTGF*, 5'-CTTGCGAAGCTGACCTGGAAGA-3' (forward) and 5'-CCGTCGGTACATACTCCACAGA-3' (reverse); *GAPDH*, 5′-ACCACAGTCCATGCATCAC-3′ (forward) and 5′-TCCACCACCCTGTTGCTGTA-3′ (reverse).

In vitro PARP-1 assay

PARP-1 activity of TI-*12403* and XAV939 was measured using a PARP Universal Colorimetric Assay Kit (Trevigen, Gaithersburg, MD, USA) according to the manufacturer's protocol.

CYP450 assay

An incubation mixture was prepared with human liver microsomes (0.25 mg/mL), 0.1 M phosphate buffer (pH 7.4), and substrates of five metabolites (50 μM phenacetin, 10 μM diclofenac, 100 μM S-mephenytoin, 5 μM dextromethorphan, and 2.5 μM midazolam). TI-12403 (0 and 10 μM) was added

to the mixture and incubated at 37 °C for 5 min. NADPH-generating system solution was then added, followed by incubation at 37 °C for 15 min. Next, to terminate the reaction, acetonitrile solution containing an internal standard (terfenadine) was added and centrifuged for 5 min (14,000 rpm, 4 °C), and the supernatant was injected into the LC-MS/MS system.

Plasma stability assay

Plasma from humans and rats was placed in tubes containing 10 μM TI-12403 and then incubated at 37 °C for the indicated time (0, 30, and 120 min). Then, acetonitrile solution containing an internal standard (chlopropamide) was placed into a tube containing plasma, vortexed for 5 min, centrifuged for 5 min (14,000 rpm, 4 °C), and then injected into the LC-MS/MS column.

Liver microsome stability assay

TI-12403 (1 μM) was added to human, rat, and mouse liver microsomes (0.5 mg/mL) in 0.1 M phosphate buffer (pH 7.4) and incubated at 37 °C for 5 min. Then, the NADPH regeneration system solution was added and incubated for 30 min at 37 °C. Next, to terminate the reaction, acetonitrile solution containing an internal standard (chlorpropamide) was added and centrifuged for 5 min (14,000 rpm, 4 °C), and the supernatant was injected into an LC-MS/MS system.

LC-MS/MS analysis

Metabolites of each CYP isozyme indicator drug that was generated through the above reaction were analyzed using the Shimadzu Nexera XR system and TSQ vantage (Thermo). A Kinetex C18 column (2.1 × 100 mm, 2.6 μm particle size, Phenomenex, Torrance, CA, USA) was used for HPLC. The mobile phases comprised distilled water containing 0.1% formic acid (A) and acetonitrile (B) containing 0.1% formic acid, and the following gradient program was used:

Time (min)	Flow (mL/min)	% A	% B
0	0.3	100	0
1.0	0.3	60	40
4.0	0.3	50	50
4.1	0.3	100	0
7.0	0.3	100	0

The generated metabolites were quantified using multiple reaction monitoring (MRM) quantification mode, and Xcalibur (version 1.6.1) was used for data analysis.

Supplementary Schemes.

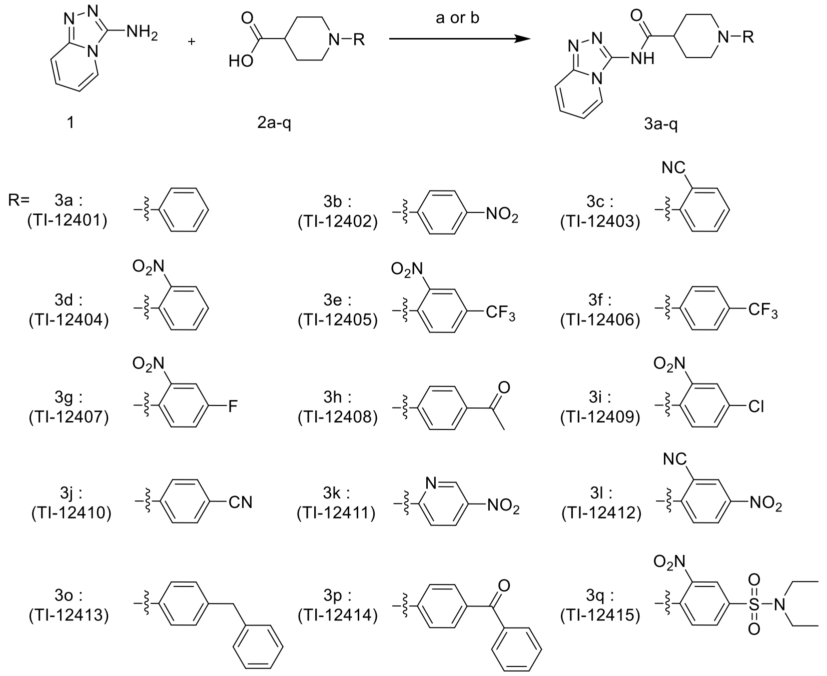

Supplementary Scheme S1. General synthetic scheme for preparation of triazololpyridyl analogues; (a) EDCl and HOBt in the presence of trimethylamine and the solvent dichloromethane at room temperature. (b) CH_2Cl_2 and HATU in the presence of trimethylamine at room temperature.

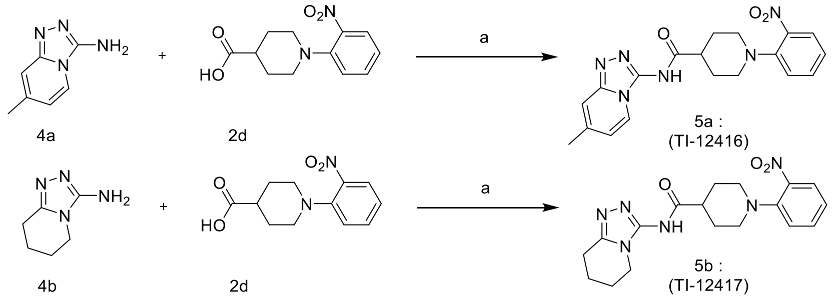

Supplementary Scheme S2. Synthetic scheme for preparation of triazololpyridine or tetrahydro-triazolopyridine analogues; (a) CH_2Cl_2 and HATU in the presence of trimethylamine at room temperature.

Supplementary Table S1. Inhibitory activities (%) of 17 synthesized compounds (1 μM concentration) on TNKS1 and TNKS2.

Compounds	Structure	TNKS1	TNKS2
TI-12401	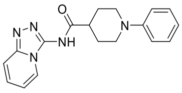	86 ± 0.5	80 ± 2.3
TI-12402	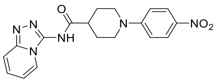	97 ± 0.5	81 ± 4.9
TI-12403	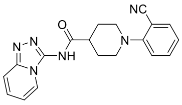	100 ± 0	93 ± 0.7
TI-12404	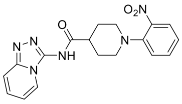	99 ± 0	90 ± 7.0
TI-12405	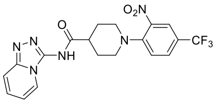	100 ± 0	96 ± 1.1
TI-12406	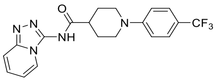	96 ± 0	93 ± 5.1
TI-12407	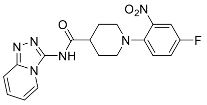	100 ± 0	86 ± 0.9
TI-12408	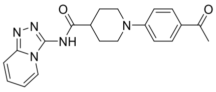	98 ± 0	98 ± 3.2
TI-12409	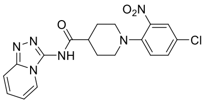	100 ± 0	92 ± 2.5
TI-12410	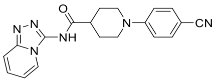	100 ± 0	86 ± 1.5
TI-12411	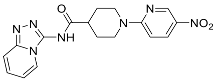	99 ± 0	96 ± 0.5
TI-12412	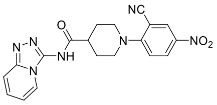	100 ± 0	91 ± 1.0
TI-12413	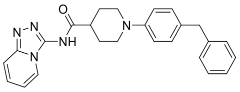	22 ± 1	35 ± 0.8
TI-12414	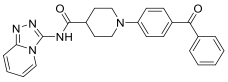	8 ± 1	32 ± 1.0
TI-12415	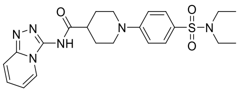	16 ± 0.5	61 ± 1.6
TI-12416	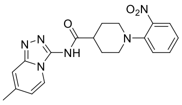	24 ± 1	60 ± 1.0
TI-12417	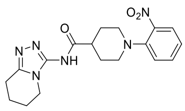	100 ± 0	96 ± 0.2
XAV939	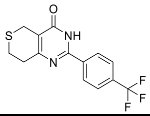	100 ± 0	99 ± 0.7

Supplementary Table S2. Inhibitory activities (%) of TI-12403 on PARP-1.

**Compound** (10 μM)	**PARP-1**
TI-12403	7 ± 0.8
XAV939	76 ± 0.3

Supplementary Table S3. CYP isozyme activity and liver microsome and plasma stability of TI-12403.

	**Contents**	**TI-12403**
CYP isozyme activity	CYP1A2	95.3
	CYP2C9	> 100
	CYP2C19	94.2
	CYP2D6	95.4
	CYP3A4	98.1
Liver microsome stability	Human	> 100
	Rat	47.7
	Mouse	50.3
Plasma stability	Human	90.7 (30 min)
		97.1 (120 min)
	Rat	97.9 (30 min)
		74 (120 min)

Supplementary Figures.

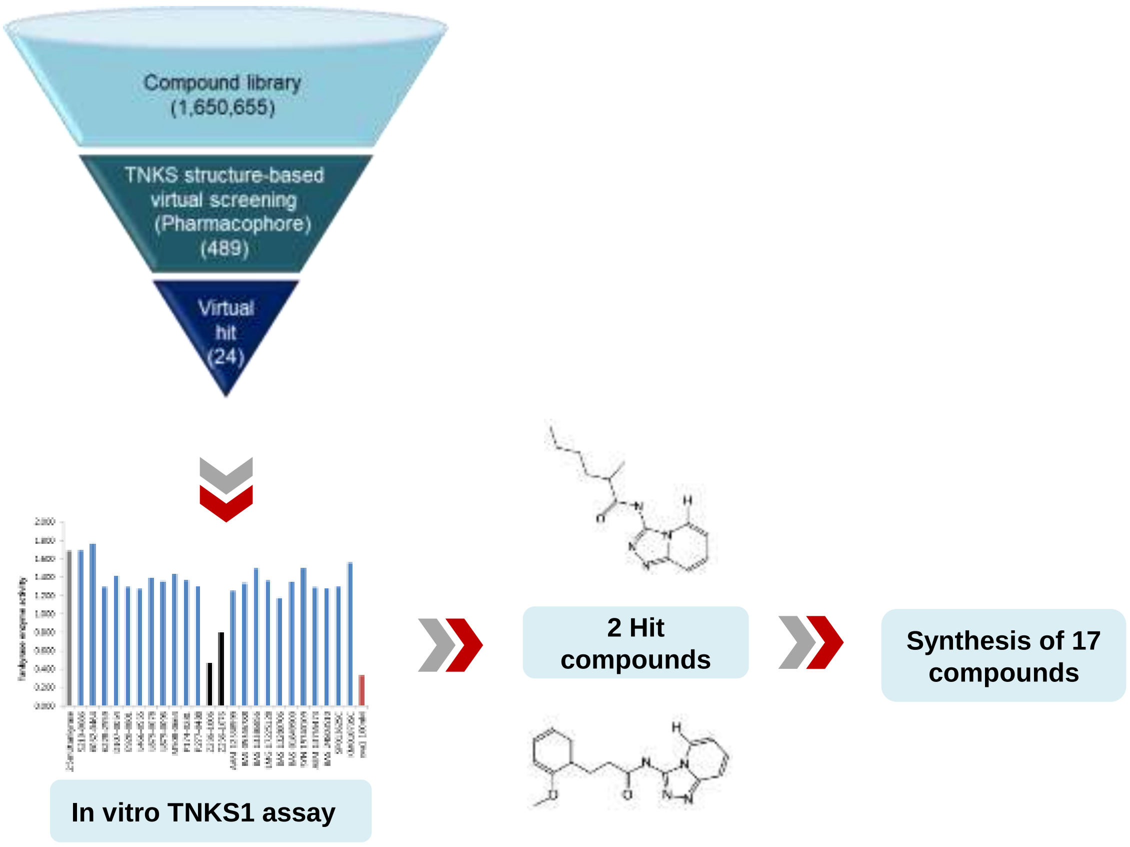

Supplementary Figure S1. Compound design and synthesis using the crystal structure obtained in the TNKS1-based virtual screening. We found 24 virtual hits through in silico and virtual screening of 1,650,655 compounds. Then, two hit compounds were discovered through an in vitro TNKS1 enzyme assay. Based on these compounds, 17 compounds were designed and synthesized.

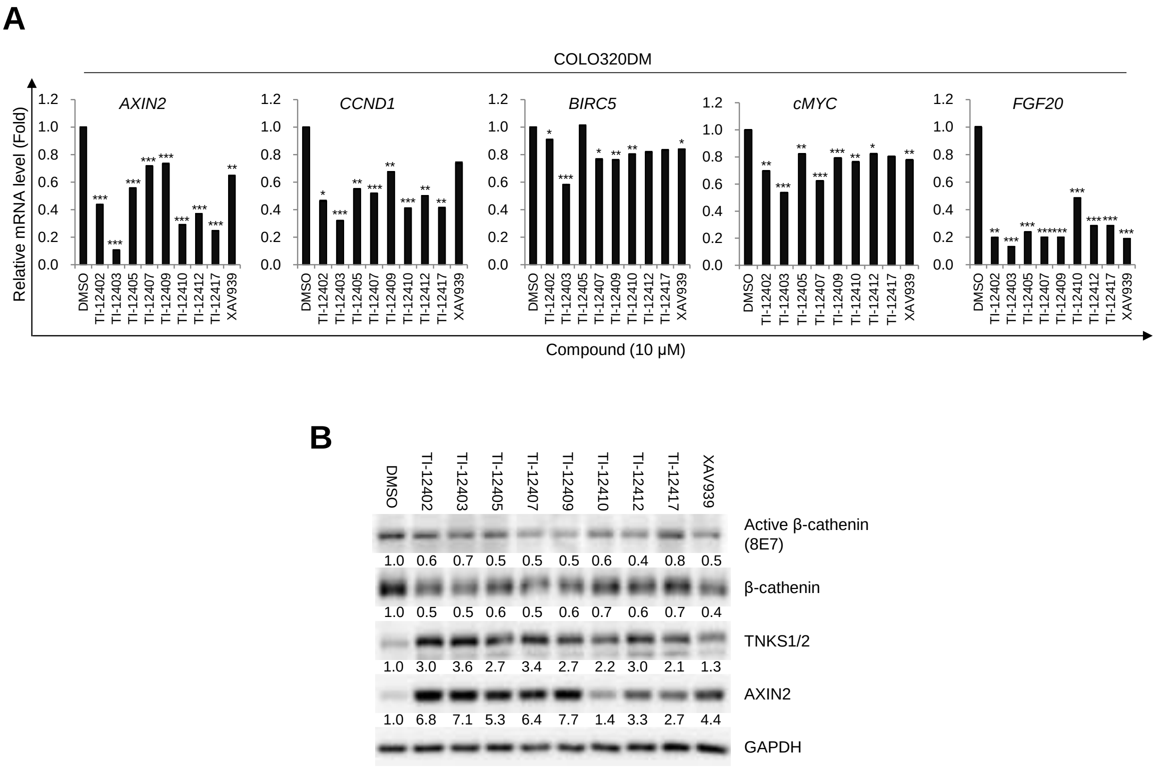

Supplementary Figure S2. Screening for compounds with inhibitory effect on β-catenin signaling in COLO320DM cells. (A and B) Human colorectal cancer (CRC) COLO320DM cells were treated with 10 μM of each TI compound for 24 h. (A) mRNA expression levels of the indicated β-catenin target genes were quantified using quantitative polymerase chain reaction (qPCR). Data represent the mean ± standard deviation (SD) of three independent experiments. *$p < 0.05$, **$p < 0.01$, ***$p < 0.001$ versus respective DMSO-treated cells. (B) Whole cell lysates were subjected to immunoblotting for detection of active β-catenin (ABC), total β-catenin, tankyrase 1/2 (TNKS1/2), and AXIN2. GAPDH was used as a loading control. The density of each band was measured by Image J software and normalized to that of GAPDH. Data represent the mean ± SD of three independent experiments. *$p < 0.05$, **$p < 0.01$ versus respective DMSO-treated cells.

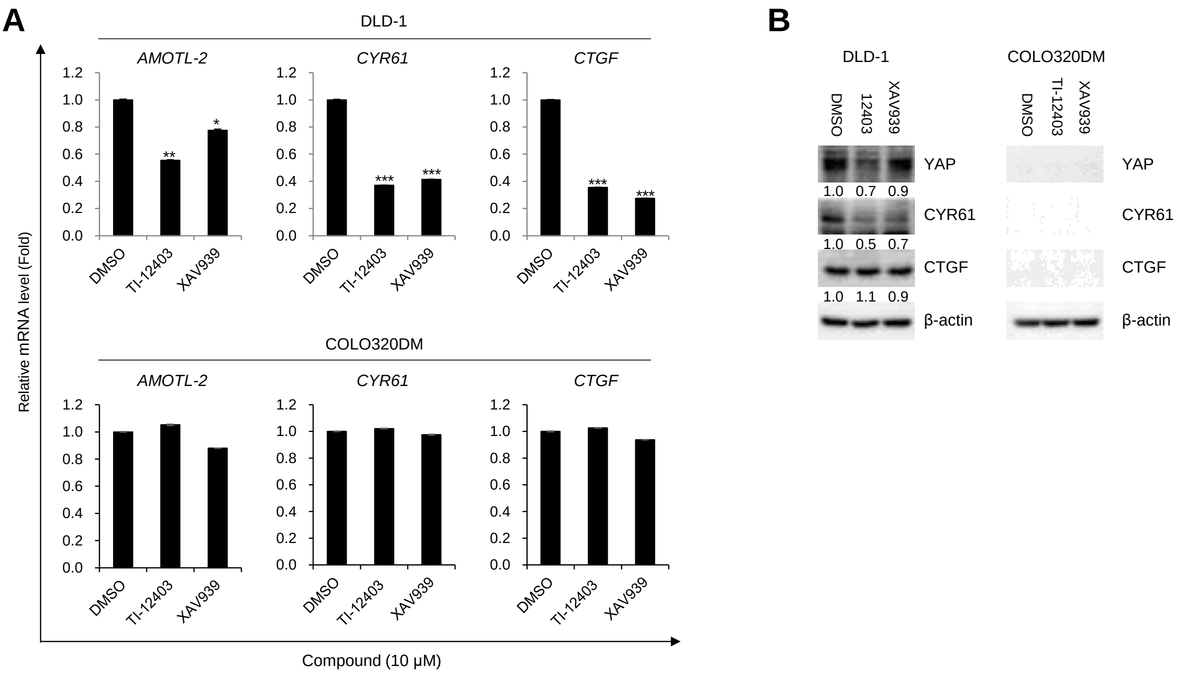

Supplementary Figure S3. TI-12403 inhibited YAP expression in DLD-1 cells. Human CRC DLD-1 cells were treated with 10 μM TI-12403 for 24 h. (A) mRNA levels of YAP target genes (*AMOTL-2*, *CYR61,* and *CTGF*) were measured by quantitative polymerase chain reaction (qPCR). Data are from one representative experiment performed in triplicate. Significant differences are defined as *$p < 0.05$, **$p < 0.01$, and ***$p < 0.001$. (B) Whole cell lysates were subjected to immunoblotting for detection of YAP, CYR61, and CTGF. β-actin was used as a loading control.
